# Evaluation of different storage times and preservation methods on phlebotomine sand fly DNA concentration and purity

**DOI:** 10.1186/s13071-020-04270-4

**Published:** 2020-08-06

**Authors:** Kamila Gaudêncio da Silva Sales, Débora Elienai de Oliveira Miranda, Fernando José da Silva, Domenico Otranto, Luciana Aguiar Figueredo, Filipe Dantas-Torres

**Affiliations:** 1 Department of Immunology, Aggeu Magalhães Institute, Oswaldo Cruz Foundation (Fiocruz), Recife, Brazil; 2 Department of Veterinary Medicine, University of Bari, Valenzano, Italy; 3 Faculty of Veterinary Sciences, Bu-Ali Sina University, Hamedan, Iran

**Keywords:** Phlebotomine, Preservation, Ethanol, DNA, *cox*1, Cacophony

## Abstract

**Background:**

Different methods have been used to preserve phlebotomine sand flies for research purposes, including for taxonomic studies and detection of *Leishmania* spp. Here, we evaluated the effect of various preservation methods at different storage times on phlebotomine sand fly DNA concentration and purity.

**Methods:**

Field-collected phlebotomine sand flies were individually stored in 70% ethanol (G1) and 95% ethanol (G2) at room temperature, 70% ethanol (G3) and 95% ethanol (G4) at 8 °C or frozen dry (i.e. no preservation solution) at − 20 °C (G5). DNA concentration and purity were assessed at various storage times (T1, ≤ 12 h; T2, 3 months; T3, 6 months; T4, 9 months; and T5, 12 months). Fragments of the cytochrome *c* oxidase subunit 1 (*cox*1) and cacophony (*CAC*) genes of phlebotomine sand flies were also amplified.

**Results:**

Mean DNA concentration (*P* = 0.178) and 260/280 purity ratios (*P* = 0.584) did not vary significantly among various preservation methods and storage times. Within each group, DNA concentration varied in G1 (Kruskal-Wallis H-test, *P* = 0.009) for T3 *vs* T4 (Dunn’s *post-hoc*, *P* < 0.05), and in G2 (Kruskal-Wallis H-test, *P* = 0.004) for T1 *vs* T2 and T1 *vs* T4 (Dunn’s *post-hoc*, *P* < 0.05). For 260/280 purity ratios, the only statistically significant difference was found for G5 (Kruskal-Wallis H-test, *P* = 0.020) between T1 *vs* T4 (Dunn’s *post-hoc* test, *P* < 0.05). The *cox*1 and *CAC* genes were successfully amplified, regardless of the preservation method and storage time; except in one sample from G2 at T1, for which the *CAC* gene failed to amplify.

**Conclusions:**

The preservation methods and storage times herein evaluated did not affect the concentration and purity of DNA samples obtained from field-collected phlebotomine sand flies, for up to 12 months. Furthermore, these preservation methods did not interfere with PCR amplification of *CAC* and *cox*1 genes, being suitable for molecular analyses under the conditions studied herein.
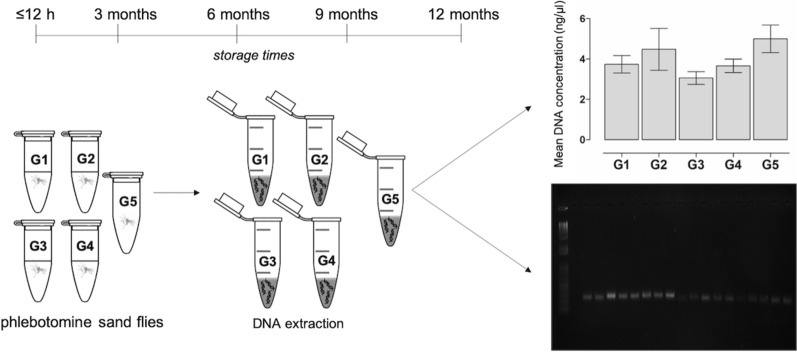

## Background

Phlebotomine sand flies are dipterans of medical and veterinary significance, due to their ability to transmit disease agents of various animal species, including humans [1]. While they also transmit viruses and bacteria, they are mostly known as biological vectors of *Leishmania* spp. parasites, which cause approximately 0.2–0.4 million cases of visceral leishmaniasis and 0.7–1.2 million cases of cutaneous leishmaniasis, in 98 countries every year [2].

Phlebotomine sand flies inhabit various types of environments, including caves, forests, crop plantations and human houses [3]. Some of these environments are difficult to access and far from research centres, making their transportation and preservation a critical step in research projects focused on the biology, taxonomy, genetics, and vector role of these insects.

Preservation methods for phlebotomine sand flies depend on the purpose of the research [4]. For studies involving DNA amplification and, eventually, DNA sequencing, they can be frozen dry at − 20 °C, frozen in liquid nitrogen at − 80 °C, or preserved in ethanol 70–100% or in dimethyl sulfoxide or in silica gel [3, 5–8]. However, each of these strategies has advantages and disadvantages in terms of preservation, practicality, and overall costs.

The use of 70% ethanol for preserving field-collected insects has become very popular in phlebotomine sand fly research [9, 10], though potential disadvantages (e.g. evaporation of ethanol and deterioration of specimens) have been acknowledged. In addition, the long-term use of 70% ethanol may also impair a correct identification of specimens by hardening the muscles of the insects and obscuring internal structures (e.g. female spermathecae) that are of prime taxonomic interest [6, 10]. Furthermore, 70% ethanol has been suggested as not an optimal preservation method for subsequent molecular analysis [11–13]. In this perspective, some authors prefer to preserve female phlebotomine sand flies in dimethyl sulfoxide for subsequent detection of *Leishmania* DNA [14, 15], but this method is more expensive than ethanol [16].

The potential deleterious effects of 70% ethanol on the concentration and purity of DNA of arthropods of medical and veterinary importance, including sand flies, is poorly investigated [11, 17, 18]. In this context, we evaluated the effect of various preservation methods involving the use of ethanol on the concentration and purity of phlebotomine sand fly DNA for up to 12 months.

## Methods

### Phlebotomine sand fly collection

Phlebotomine sand fly collections were carried out in the municipalities of Pesqueira (8°21′35″S, 36°41′42″W) and Machados (7°40′56″S, 35°31′22″W), which are located in the Agreste region of Pernambuco State, Brazil [19, 20]. Four CDC light traps were installed in each municipality in two consecutive days of February 2018, operating from 18:00 h to 6:00 h of the next day. Each trap was positioned at 1.5 m above the ground level, outside human houses. After the capture, the nets were detached from the traps and placed in plastic bags containing cotton wool soaked in chloroform (~2 ml) for 20 min to kill the insects. Immediately, phlebotomine sand flies were separated from the other insects under a stereomicroscope and stored according to the preservation methods described below, before being transported to the laboratory on the same day.

Phlebotomine sand flies used herein were not identified to species level, in order to avoid excessive manipulation of the specimens. Indeed, cutting of parts of the phlebotomine sand flies (e.g. head and last abdominal segments of females) would potentially introduce a bias in terms of size variability, which could ultimately result in significant differences in DNA concentration and purity.

### Preservation of phlebotomine sand flies

Field-collected phlebotomine sand flies (*n* = 250; 125 from Pesqueira and 125 from Machados) were individually stored using five different preservation methods and for different storage times. In particular, each group was composed of 10 phlebotomine sand flies (regardless the sex), which were individually placed into 1.5 ml sterile tubes with 70% ethanol (G1) and 95% ethanol (G2) at room temperature, 70% ethanol (G3) and 95% ethanol (G4) at 8 °C or frozen dry (i.e. no preservation solution) at − 20 °C (G5). The different concentrations of ethanol were made by diluting 98.8% ethanol (Neon commercial-03467; São Paulo, Brazil) with sterile distilled water.

Preservation of phlebotomine sand flies lasted for variable storage times (T): T1, < 12 h; T2, 3 months; T3, 6 months; T4, 9 months; and T5, 12 months. For T1, DNA extraction (described below) was done in same collection day, whereas for T2 to T5 it was performed at the end of each storage time.

### DNA extraction and assessment

DNA extraction from phlebotomine sand flies was performed according to storage times using DNeasy Blood & Tissue kit (Qiagen GmbH, Hilden, Germany), following the manufacturer’s instructions. All samples were eluted in 200 µl of Buffer AE (10mM Tris Cl, 0.5 mM EDTA, pH 9.0), and labelled with information about the group and storage time. DNA concentration and the ratio of the absorbance at 260 and 280 nm (A_260/280_ ratio) were evaluated in the same day of DNA extraction using a NanoDrop Lite spectrophotometer (Thermo Fisher Scientific, Waltham, USA), with 1 µl of DNA extraction loaded directly on the optical surface. After DNA extraction, all samples were stored at − 20 °C until testing.

### PCR amplification

The DNA integrity was further assessed by PCR using the primers 5Llcac (5′-GTG GCC GAA CAT AAT GTT AG-3′) and 3Llcac (5′-CCA CGA ACA AGT TCA ACA TC-3′), which amplify a 220-bp fragment of the cacophony gene (*CAC*) of phlebotomine sand flies [21, 22] and the primers LCO1490 (5′-GGT CAA CAA ATC ATA AAG ATA TTG G-3′) and HC02198 (5′-TAA ACT TCA GGG TGA CCA AAA AAT CA-3′), which amplify a ~658-bp fragment of the mitochondrial cytochrome *c* oxidase subunit 1 gene (*cox*1) of invertebrates [23]. These genes are constitutively expressed in phlebotomine sand flies and were used as internal controls. They are also commonly used in molecular systematics of phlebotomine sand flies [24].

All PCR reactions were performed in a final volume of 25 µl containing 8.5 µl of DNA-free water, 12.5 µl of GoTaq^™^ Colorless Master Mix, 1 µl of each primer at a concentration of 12.5 pmol/µl and 2 µl sample DNA. A master mix without DNA (no template control, NTC) was included in all reactions. Additionally, DNA samples extracted from phlebotomine sand flies stored for ≤ 12 h (T1) were used as a positive control and to test for primer efficiency. For the *CAC* gene, PCR thermal conditions included a denaturation step at 95 °C for 2 min, followed by 30 cycles of 95 °C for 30 s, 60 °C for 30 s, 72 °C for 30 s, with a final extension step at 72 °C for 5 min. For *cox*1 gene, PCR thermal conditions were as follows: initial denaturation at 95 °C for 3 min, followed by 37 cycles for 95 °C for 1 min, 50 °C for 1 min, 72 °C for 7 min, with a final extension step at 72 °C for 7 min.

After PCR, 5 µl of PCR products stained with ethidium bromide (10 mg/ml) were loaded on 1.5% agarose gel and visualized using a UV light. Amplification was considered successful when a single band of the expected size was visualised.

### Data analysis

Normality of data was assessed using Lilliefors. Then, Kruskal-Wallis H-test (with Dunn’s *post-hoc* test) was used to verify the differences in DNA concentration and purity among preservation methods and storage times. Statistical analyses were performed using BioEstat v.5.3 [25] and *P* ≤ 0.05 was considered statistically significant.

## Results

**Fig. 1:**
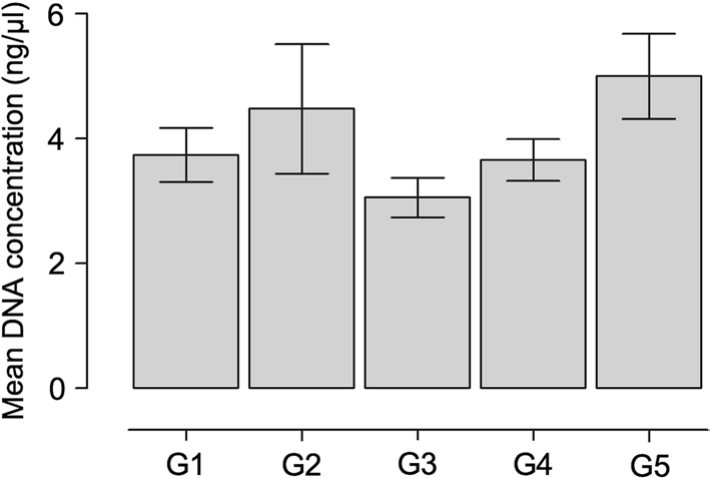
Mean DNA concentration (ng/µl) obtained from phlebotomine sand flies according to different preservation methods. DNA concentration was evaluated in the same day of DNA extraction. Preservation methods include 70% ethanol (G1) and 95% ethanol (G2) at room temperature, 70% ethanol (G3) and 95% ethanol (G4) at 8 °C, and frozen dry at − 20 °C (G5)

Mean DNA concentrations for all preservation methods (G1 to G5) and store times (T1 to T5) are depicted in Table 1. The highest mean DNA concentration was found in G2 and the lowest in G3, with DNA concentrations ranging from 2.7–8.2 ng/µl and 2.3–4.2 ng/µl, respectively, depending on storage time (Fig. 1). The A_260/280_ ratio obtained between the preservation methods ranged from 1.6–2.1. There was no significant difference between the different groups in terms of mean DNA concentration (Kruskal-Wallis H-test, *H* = 6.30, *df* = 4, *P* = 0.178) and mean A_260/280_ values (Kruskal-Wallis H-test, *H* = 2.85, *df* = 4, *P* = 0.584) in function of storage time.

**Table 1:** Mean concentration (ng/µl) and 260/280 purity ratios of DNA extracts obtained from phlebotomine sand flies according to different preservation methods and storage times. PCR success (positive/total and percentage) for the mitochondrial cytochrome *c* oxidase subunit 1 (*cox*1) and cacophony (*CAC*) genes is also shown

Preservation method	Storage time	DNA concentration (mean ± SD)	260/280 ratio (mean ± SD)	Success rate of PCR			
				*CAC* gene	%	*cox*1 gene	%
70% ethanol at room temperature (G1)	T1	4.5 ± 1.8	1.8 ± 0.4	10/10	100	10/10	100
	T2	3.0 ± 0.9	2.0 ± 0.2	10/10	100	10/10	100
	T3	4.9 ± 2.2	1.9 ± 0.2	10/10	100	10/10	100
	T4	2.6 ± 1.4	1.9 ± 0.1	10/10	100	10/10	100
	T5	3.7 ± 1.1	1.9 ± 0.2	10/10	100	10/10	100
95% ethanol at room temperature (G2)	T1	8.2 ± 5.7	1.7 ± 0.5	9/10	90	10/10	100
	T2	2.7 ± 0.6	1.9 ± 0.2	10/10	100	10/10	100
	T3	5.2 ± 5.7	1.8 ± 0.2	10/10	100	10/10	100
	T4	2.7 ± 0.5	2.0 ± 0.2	10/10	100	10/10	100
	T5	3.6 ± 0.7	2.0 ± 0.3	10/10	100	10/10	100
70% ethanol at 8 °C (G3)	T1	4.2 ± 2.7	1.8 ± 0.2	10/10	100	10/10	100
	T2	2.3 ± 1.5	1.9 ± 0.2	10/10	100	10/10	100
	T3	3.1 ± 0.9	1.9 ± 0.3	10/10	100	10/10	100
	T4	2.7 ± 1.1	1.9 ± 0.2	10/10	100	10/10	100
	T5	3.0 ± 0.7	2.1 ± 0.2	10/10	100	10/10	100
95% ethanol at 8 °C (G4)	T1	4.9 ± 3.1	1.8 ± 0.4	10/10	100	10/10	100
	T2	3.2 ± 0.5	1.8 ± 0.2	10/10	100	10/10	100
	T3	3.8 ± 1.2	1.9 ± 0.2	10/10	100	10/10	100
	T4	3.1 ± 1.2	1.9 ± 0.2	10/10	100	10/10	100
	T5	3.3 ± 1.3	2.0 ± 0.3	10/10	100	10/10	100
Frozen dry at − 20 °C (G5)	T1	7.7 ± 3.8	1.6 ± 0.3	10/10	100	10/10	100
	T2	4.0 ± 1.8	1.8 ± 0.2	10/10	100	10/10	100
	T3	4.5 ± 2.2	1.8 ± 0.2	10/10	100	10/10	100
	T4	4.5 ± 2.4	1.9 ± 0.2	10/10	100	10/10	100
	T5	4.3 ± 0.9	1.9 ± 0.2	10/10	100	10/10	100

*Abbreviations*: T1, < 12 h; T2, 3 months; T3, 6 months; T4, 9 months; T5, 12 months

Considering each individual group, DNA concentrations obtained in G1 varied significantly (Kruskal-Wallis H-test, *H* = 13.45, *df* = 4, *P* = 0.009) for T3 *vs* T4 (Dunn’s *post-hoc*, *P* < 0.05). In the same way, DNA concentrations obtained in G2 varied significantly (Kruskal-Wallis H-test, *H* = 15.30, *df* = 4, *P* = 0.004) for T1 *vs* T2 and T1 *vs* T4 (Dunn’s *post-hoc*, *P* < 0.05). No differences were found in the DNA concentrations obtained in G3 (Kruskal-Wallis H-test, *H* = 7.20, *df* = 4, *P* = 0.126), G4 (Kruskal-Wallis H-test, *H* = 4.94, *df* = 4, *P* = 0.293) and G5 (Kruskal-Wallis H-test, *H* = 8.71, *df* = 4, *P* = 0.069) in function of storage time.

For A_260/280_ ratios, the only statistically significant difference was found in G5 (Kruskal- Wallis H-test, *H* = 11.63, *df* = 4, *P* = 0.020; Dunn’s *post-hoc*, *P* < 0.05) for T1 *vs* T4. No significant difference was found for G1 (Kruskal-Wallis H-test, *H* = 5.45, *df* = 4, *P* = 0.245), G2 (Kruskal-Wallis H-test, *H* = 7.06, *df* = 4, *P* = 0.133), G3 (Kruskal-Wallis H-test, *H* = 7.68, *df* = 4, *P* = 0.104) and G4 (Kruskal-Wallis H-test, *H* = 4.28, *df* = 4, *P* = 0.369).

PCR amplification of the *cox*1 and *CAC* genes was successful, regardless the preservation method and the storage time. Of the 250 DNA extracts, only one sample (from G2 at T1: concentration of 3.9 ng/µl; A_260/280_ ratio of 1.9) failed to amplify the *CAC* gene fragment.

## Discussion

None of the preservation methods assessed in the current study significantly affected the concentration and the purity of DNA samples obtained from field-collected phlebotomine sand flies. These results confirm that all preservation methods investigated herein are suitable for phlebotomine sand fly research and indicate that the decision of which method to use should be a matter of convenience. For instance, 70% ethanol at room temperature may be the most convenient method for researchers working in remote areas, far away from a laboratory structure. Furthermore, phlebotomine sand flies used herein were preserved individually, which is a good practice compared to storing all specimens in a single vial. This would be particularly recommended for insects that are designated for molecular analysis.

Considering that phlebotomine sand flies are small, one of the main technical challenges for molecular studies is to isolate enough DNA [26]. Specimens kept frozen dry have been used successfully for different approaches, such as mitochondrial genes amplification and sequencing for population genetics [27] and species identification by matrix-assisted laser desorption/ionization time of flight mass spectrometry (MALDI-TOF MS) [28, 29]. It has been suggested that freezing specimens without the use of a preservation agent should have a lower potential for introducing contaminants than using reagents [30]. However, freezing is generally impractical under field conditions, also making sample transportation a difficult task.

Traditionally, in large-scale field studies collected specimens are often killed and preserved in ethanol, prior to DNA extraction [3, 9]. Ethanol is generally suitable for DNA analysis [31] and it has been suggested that 99% ethanol can also be used for molecular studies allowing a viral genome RNA identification [4]. While the long-term use of 70% ethanol may eventually affect the identification phlebotomine sand flies [6, 10], in our experience, this is not usually a problem when examining slide-mounted specimens that have been preserved in 70% ethanol at room temperature for some months or even a year. Another limitation of 70% ethanol is that it cannot be employed for preserving samples for isoenzyme analysis [31]. In this case, phlebotomine sand flies should be kept alive, stored at temperatures below − 40 °C (e.g. − 80 °C) or cryopreserved [31, 32].

The storage time had no negative effect on the DNA concentration in any of the preservation methods used herein. In addition, the integrity of the extracted DNA at all storage times was confirmed by the amplification of *CAC* and *cox*1 genes by PCR in 99.6% of the cases; only one out of 250 DNA extracts failed to amplify the *CAC* gene (Table 1), which suggests that DNA extraction was not successful for this sample. More recently, a fast multiplex real-time PCR assay for simultaneous detection of blood meals and *Leishmania* parasites in female phlebotomine sand flies demonstrated promising results using specimens that were stored in 70% ethanol for approximately two years [20, 33]. Interestingly, female phlebotomine sand flies stored at − 20 °C for approximately four years were successfully used to detect host DNA through real-time PCR assays [34]. These data show that these storage methods were suitable for host DNA and *Leishmania* spp. detection in phlebotomine sand flies. However, it is known that depending on storage conditions, samples can deteriorate over time [13]. Hence, the correct choice of the preservation method can guarantee the possibility of obtaining successful results after sampling, avoiding losing the overall quality of the samples for certain types of studies.

## Conclusions

Our results show that all preservation methods assessed, including 70% ethanol at room temperature, did not affect significantly the purity and concentration of DNA samples obtained from field-collected phlebotomine sand flies. Moreover, these preservation methods did not interfere with PCR amplification of *CAC* and *cox*1 genes. Further research is indicated to evaluate if these methods may interfere with the amplification of microorganisms in phlebotomine sand flies, including those part of the sand fly microbiota and also *Leishmania* parasites.

## Data Availability

The data supporting the conclusions of this article are included within the article. Raw data can be shared with other researchers upon a specific request.

## Abbreviations

*CAC*: cacophony gene
*cox*1: cytochrome *c* oxidase subunit 1 gene
DNA: deoxyribonucleic acid
NTC: no template control
PCR: polymerase chain reaction
